# Transcriptomics of aged *Drosophila* motor neurons reveals a matrix metalloproteinase that impairs motor function

**DOI:** 10.1111/acel.12729

**Published:** 2018-02-07

**Authors:** Jorge Azpurua, Rebekah E. Mahoney, Benjamin A. Eaton

**Affiliations:** ^1^ Department of Anesthesiology Stony Brook University School of Medicine Stony Brook NY USA; ^2^ Department of Cellular and Integrative Physiology UTHSCSA San Antonio TX USA; ^3^ Barshop Institute for Longevity and Aging Studies UTHSCSA San Antonio TX USA

**Keywords:** aging, matrix metalloproteinase, MMP1, motor neuron, TDP43, TIMP, transcriptomics

## Abstract

The neuromuscular junction (NMJ) is responsible for transforming nervous system signals into motor behavior and locomotion. In the fruit fly *Drosophila melanogaster*, an age‐dependent decline in motor function occurs, analogous to the decline experienced in mice, humans, and other mammals. The molecular and cellular underpinnings of this decline are still poorly understood. By specifically profiling the transcriptome of *Drosophila* motor neurons across age using custom microarrays, we found that the expression of the matrix metalloproteinase 1 (*dMMP1*) gene reproducibly increased in motor neurons in an age‐dependent manner. Modulation of physiological aging also altered the rate of dMMP1 expression, validating *dMMP1* expression as a bona fide aging biomarker for motor neurons. Temporally controlled overexpression of *dMMP1* specifically in motor neurons was sufficient to induce deficits in climbing behavior and cause a decrease in neurotransmitter release at neuromuscular synapses. These deficits were reversible if the *dMMP1* expression was shut off again immediately after the onset of motor dysfunction. Additionally, repression of dMMP1 enzymatic activity via overexpression of a tissue inhibitor of metalloproteinases delayed the onset of age‐dependent motor dysfunction. MMPs are required for proper tissue architecture during development. Our results support the idea that matrix metalloproteinase 1 is acting as a downstream effector of antagonistic pleiotropy in motor neurons and is necessary for proper development, but deleterious when reactivated at an advanced age.

## 1 INTRODUCTION

The aging of the nervous system is manifested not only in the decline of cognition and memory, but also in the progressive decline of motor coordination and muscle function (Doherty, 2003). These declines in motor function have been observed throughout the animal kingdom including in humans, rodents, and insects (Jang & Van Remmen, 2011; Seidler et al., 2010) (Gargano et al., 2005; Kreko‐Pierce et al., 2016). In mammals, much of the motor decline is due to muscle fiber loss and declining fiber contractility, but altered motor neuron (MN) function also contributes (Edstrom et al., 2007). Previous work suggests that neurons are particularly vulnerable to age‐accumulated damage from reactive oxygen species (ROS), misfolded proteins, and inflammation, but the contribution of these mechanisms to declining motor function remains to be established (Lin & Beal, 2006; Saxena & Caroni, 2011). Aging is also the most significant risk factor in a wide array of neurodegenerative disorders, including Alzheimer's disease (AD), Parkinson's disease (PD), and amyotrophic lateral sclerosis (ALS) (Armon, 2003; Herbert et al., 2001; Levy et al., 2002). A perplexing feature of all of these diseases is that there exist high‐risk alleles and lesions that predispose carriers to neurodegeneration that are present from conception, yet disease pathology only manifests in mid‐ or late life after interaction with the normal aging process (or by altering its basal rate). Thus, the molecular mechanisms that determine the pathogenesis of these disorders are not only tissue‐specific (neurons) but also temporally constrained (old age). How aging affects postmitotic MNs resulting in impaired motor function and increased susceptibility to disease is poorly understood.

In order to elucidate the molecular events that comprise neuronal aging and allow neurodegenerative disorders to manifest, we developed a MN‐specific pipeline in *Drosophila melanogaster* to investigate the effect of the aging process on the MN transcriptome. In *Drosophila*, pure populations of MNs can be isolated through a combination of genetics, dissection, and cell sorting (Parrish et al., 2014). This allows for the screening of a highly homogenous population of neuronal cells, limiting confounding effects from epigenetic differences across neuronal types. MNs are highly morphologically and functionally stereotyped which allows for higher confidence in the specificity of sorting. Additionally, unlike central neurons, which may alter their gene expression in response to changing environmental circumstances, MNs have a predominantly unchanging function. We predicted this would increase the signal‐to‐noise ratio for transcriptomics across age.

Transcriptional profiling of these neurons revealed consistent age‐dependent increases in the expression of the matrix metalloproteinase 1 (*dMMP1*) gene. *dMMP1* is a member of the calcium‐dependent zinc‐containing endopeptidases of the metzincin protease superfamily (Page‐McCaw et al., 2007). MMPs play critical roles in tissue remodeling during development and have been linked to aging and neurodegeneration (Ethell & Ethell, 2007; Kiaei et al., 2007). We find that *dMMP1* expression correlates with declines in motor function and overexpression of *dMMP1* in young MNs mimics the effects of age on motor function. At the cellular level, *dMMP1* overexpression in MNs results in reduced neurotransmission. Finally, manipulations that alter the rate of aging result in altered rates of dMMP1 accumulation. These data provide evidence that dMMP1 is a biomarker of aging in *Drosophila* motor neurons that also contributes to age‐dependent motor declines.

## 2 RESULTS

### 2.1 Microarray profiling of aging drosophila motor neurons

**Figure 1:**
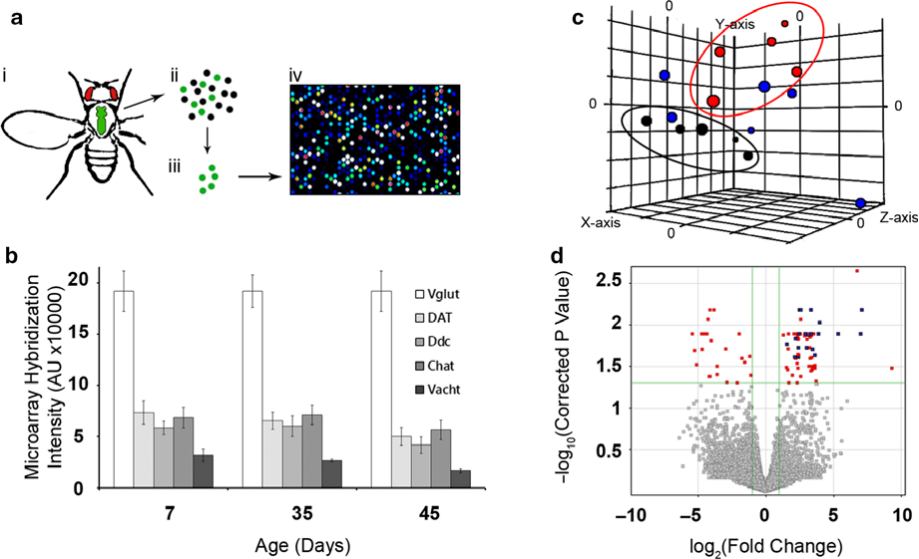
Transcriptome profiling from aging *Drosophila* motor neurons. (a) Scheme of sample preparation. (i) Lower thorax with GFP+ cells dissected. (ii) Collagenase homogenization. (iii) GFP‐positive cells are isolated via FACS. (iv) RNAs are isolated, amplified, converted to cDNA, and hybridized on microarray. (b) Neurotransmitter synthesis gene levels across age conditions. Each bar represents an average of several features representing that gene on the microarray from all fly groups of the age indicated on the *x*‐axis. (c) Principal component analysis (PCA) of individual array samples. Each colored circle on the plot indicates a sample hybridized to the microarray. Black circles—7 days old. Red circles—35 days old. Blue circles—45 days old. The elliptical shapes encircle the 7‐day and 35‐day groupings. Principal components 1, 2, and 3 are plotted on the *x*‐axis, *y*‐axis, and z‐axis. Grid scale = 1,000 a.u. (d) Volcano plot (after Benjamini–Hochberg correction for multiple testing) of 7‐day‐old vs. 45‐day‐old samples (averaged by age). Points on the right side of the plot are upregulated in the 45‐day‐old samples. Features changing by more than twofold with a *p*‐value of lower than .05 are highlighted in red. Features corresponding to the *dMMP1* gene are highlighted in blue

To discover the major changes driving MN aging in *Drosophila*, we designed custom Agilent microarrays to measure alterations in gene expression with age. The microarray was enriched with features mapping to membrane channel genes and calcium‐binding proteins (Table S1). The workflow for generating the microarray data (Figure 1a) relied on using the D42 driver to express GFP exclusively in MNs, dissect and dissociate cells from the thoracic ganglion, and isolate the GFP‐positive neurons through fluorescence‐activated cell sorting (FACS). The microarray was hybridized with doubly amplified cDNAs generated from the FACS sorted MNs. Each biologically independent replicate consisted of cDNAs derived from a pool of five age‐matched flies. Time points evaluated on our microarray were young (7 days posteclosion, *n* = 5), middle age (35 days posteclosion, *n* = 5), and post “age‐dependent potentiation” (Mahoney et al., 2014) (ADP, 45 days posteclosion, *n* = 6) flies.

We considered a possible serious confounding condition for our approach—if our D42 driver was inconsistently active across age (i.e., driving in non‐MN cells as a consequence of aging), it could lead to differential gene expression from different tissue types being mistaken for age‐driven changes. To rule out this possibility, we looked at the relative levels of neurotransmitter synthesis genes, which are highly indicative of final differentiated neuronal identity (Figure 1b). We did not find any evidence to suggest major shifts in the D42 driver cell type, and the pattern of relative expression was stable across all tested ages. We performed principal component analysis (PCA) on our dataset as part of the quality control workflow on the microarrays. We predicted that samples would cluster tightly according to age. The 7‐day‐old samples clustered together well, but older samples had more intrinsic variation and the 45‐day‐old samples did not group well (Figure 1c). This indicated that gene expression patterns might be more stable in young MNs and did not rule out the possibility that gene dysregulation was contributing to age‐dependent motor dysfunction phenotypes.

We performed several conservative statistical analyses on the finished dataset. Initially, we hypothesized we would see a large number of transcripts altered with age. Surprisingly, analysis of differentially expressed genes across all pairwise conditions (7 vs. 35, 7 vs. 45, and 35 vs. 45) after ANOVA yielded a small list of microarray features, with many mapping to the same gene (Table S2). This indicated that most genes did not change significantly across the tested ages. We focused on the transcripts that changed most robustly across age by performing a Volcano plot‐mediated *t* test between the 7‐day‐old and 45‐day‐old samples (Figure 1d). This analysis revealed 176 microarray features changing with age, corresponding to 40 unique annotated genes, of which 18 were downregulated and 21 were upregulated (Table S2).

### 2.2 dMMP1 increases with age in drosophila motor neurons

**Figure 2:**
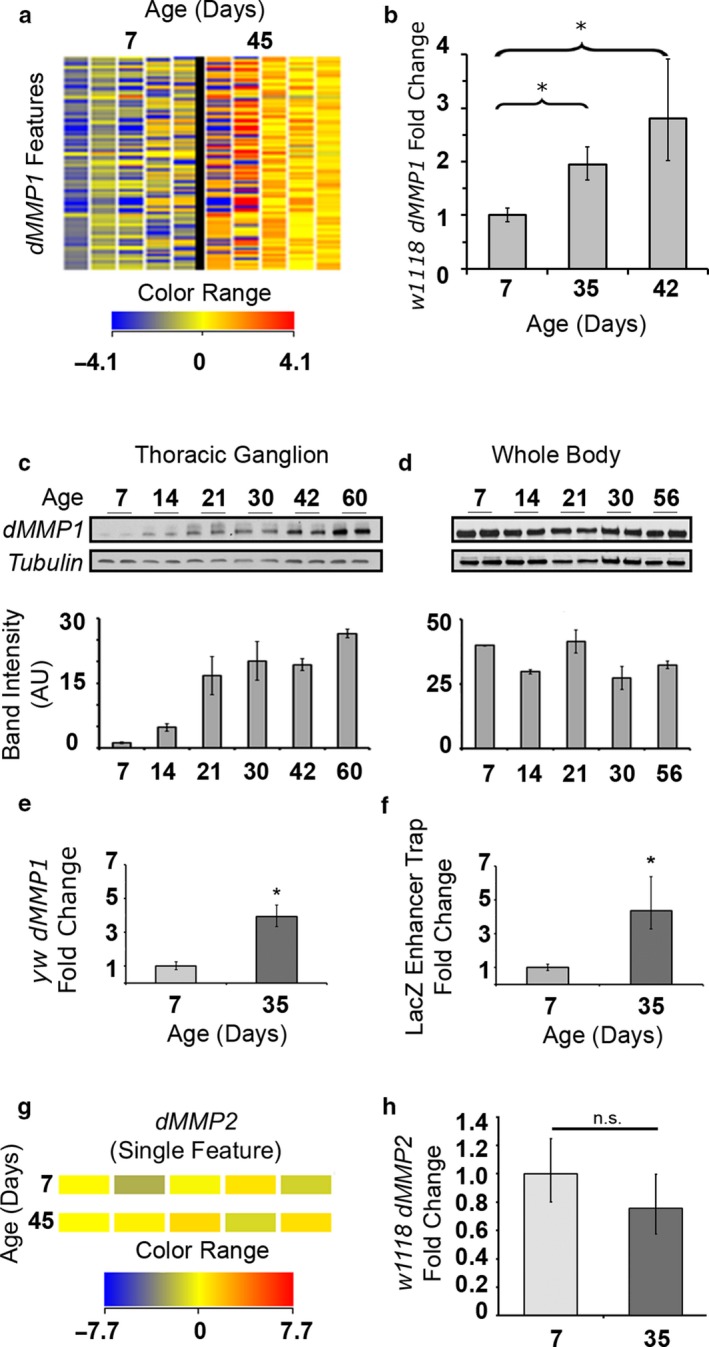
Expression profile of the *dMMP1* gene with age in motor neurons. (a) Heatmap (not averaged) of all dMMP1 features on microarray. Five 7‐day‐old samples are shown in the left column, and five 45‐day‐old samples are shown in the right column. Each row represents a feature on the microarray corresponding to *dMMP1*. More highly expressed features in the 45‐day‐old sample are shown in redder shades. The color range bar indicates how blue‐red shade corresponds to fold change expression. (b) qRT–PCR of *dMMP1* transcripts from sorted motor neuron RNA extracts. Age of sorted neurons is indicated on the *x*‐axis. Fold change was calculated relative to the 7‐day‐old sample. Error bars = 1*SD*. *p <* *.05 via Student's *t* test. (c) Western immunoblot of dMMP1 protein, thoracic ganglion extracts from flies of increasing age (tubulin loading control). Quantification of band intensity is shown below the blot, normalized to tubulin signal. Error bar = 1 *SD*. (d) Western immunoblot of dMMP1 protein, whole‐body extracts from flies of increasing age (tubulin loading control). Quantification of band intensity is shown below the blot, normalized to tubulin signal. Error bar = 1 *SD*. (e) qRT–PCR of *dMMP1* transcripts from thoracic ganglion RNA extract (yw genetic background). Age of flies is indicated in the *x*‐axis. Fold change was calculated relative to the 7‐day‐old sample. Error bars = 1*SD*. *p <* *.05 via Student's *t* test. (f) qRT–PCR of *LacZ* transcripts from thoracic ganglion RNA extracts (yw genetic background). Age of flies is indicated on the *x*‐axis. Fold change was calculated relative to the 7‐day‐old sample. Error bars = 1*SD*. *p <* *.05 via Student's *t* test. Tubulin was used as the reference gene for all qRT–PCRs. (g) Heatmap (not averaged) of the single dMMP2 feature on the microarray. (h) qRT–PCR of *dMMP2* transcripts from sorted motor neuron RNA extracts

Notably, the *dMMP1* gene was ubiquitously represented (40/40 features on the microarray, corresponding to three distinct probe sequences) in our mediated *t* test (Figure 1d), meaning that microarray features associated with *dMMP1* were consistently changing across age. A heatmap of all 40 *dMMP1* features on our microarray visually displays the change in expression across age (Figure 2a). Because of the strong and consistent signal on our arrays from all of the features of this gene, we opted to focus our downstream analysis on the role of *dMMP1* in MN aging. To verify and further expand on the results of our microarray, we performed qRT–PCR on nonamplified RNAs from FACS‐purified MNs (eliminating amplification as a potential source of artifactual results). We found that levels of the *dMMP1* transcript could also be reliably detected by qRT–PCR and that levels increased consistently in an age‐dependent manner (Figure 2b). We reasoned that increased dMMP1 transcript could have a detectable effect on the amount of dMMP1 protein in the nervous system. To test this, we dissected and isolated thoracic ganglions from adult flies of increasing age and performed Western immunoblotting of dMMP1. In concordance with the transcription data, dMMP1 protein showed a marked and consistent increase in expression (Figure 2c). Notably, dMMP1 does not show a clear increase with age in the whole body of flies (Figure 2d), and this does not rule out the possibility that *dMMP1* increases in non‐nervous system tissues, but it demonstrates it is not a general phenomenon happening in all tissues.

We also considered the possibility that *dMMP1* mRNA (and consequently protein) was accumulating due to a change in RNA stability, processing and turnover across age, rather than a result of direct transcriptional upregulation. To test this, we used a *LacZ* enhancer trap line with a mutagenic p‐element insertion in the *dMMP1* gene (Figure S2). Flies with one copy of the enhancer trap and one functional copy of dMMP1 (on the balancer chromosome) were allowed to age, and the relative transcript quantity of *dMMP1* (Figure 2e) and *LacZ* (Figure 2f) was contrasted in young and old fly thoracic ganglion by qRT–PCR. Although the genetic background of the *Drosophila* strain used for this experiment was *yellow*, the *dMMP1* increase was recapitulated, demonstrating that it is not idiosyncratic to the *w* ^*1118*^ strain used for our microarray. The *LacZ* transcript also increased in older flies supporting the interpretation that *dMMP1* message increases with age due to increased transcriptional activity downstream of the *dMMP1* promoter.

The *Drosophila* genome encodes another canonical matrix metalloproteinase, *dMMP2*, which serves similar functions as *dMMP1*. Even though this gene was not detected as changing significantly after automated statistical analysis, we directly queried our microarray data and found no evidence that dMMP2 was changing with aging in MNs with age (Figure 2g). We also tested this via qRT–PCR and again found no change in the expression of this gene (Figure 2h).

### 2.3 dMMP1 increases in response to physiological aging and pathological context

**Figure 3:**
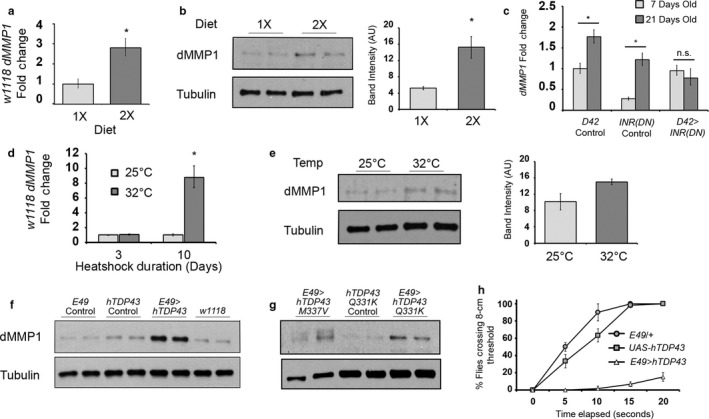
Expression profile of *dMMP1* after aging rate modulation. (a) qRT–PCR of *dMMP1* transcripts from thoracic ganglion RNA extracts (*w1118* genetic background, 14 days old). Diet is indicated on the *x*‐axis (1× and 2× yeast concentration, respectively). Fold change was calculated relative to the 1× diet sample. Error bars = 1*SD*. *p <* *.05 via Student's *t* test. (b) Western blot of dMMP1 protein and tubulin loading control from thoracic ganglion of flies on 1× and 2× diets. Quantification of band intensity is shown to the right of the blot, normalized to tubulin signal. Error bar = 1 *SD*. *p <* *.05 via Student's *t* test. (c) qRT–PCR of *dMMP1* transcripts after the abrogation of insulin signaling by *D42 > InR(DN)* in aging MNs. Sample age is indicated by bar color (light gray: 7‐day‐old, dark gray: 21‐day‐old). Fold change was calculated relative to the 7‐day‐old D42 driver control. Error bars = 1*SD*. *p <* *.05 via Student's *t* test. (d) qRT–PCR of *dMMP1* transcripts from thoracic ganglion RNA extracts (*w1118* genetic background). Left two bars are flies kept at 25°C or 32°C for a 3‐day exposure duration, and right bars are 10‐day temperature exposure duration. Fold change was calculated relative to the 25°C sample for the corresponding duration. Error bars = 1*SD*. *p <* *.05 via Student's *t* test. (e) Western blot of dMMP1 protein and tubulin loading control from thoracic ganglion of flies on 25°C or 32°C temperature exposure for 10 days. Quantification of band intensity is shown to the right of the blot, normalized to tubulin signal. Error bar = 1 *SD*. (f and g) Western blots of dMMP1 protein and tubulin loading controls from flies overexpressing hTDP43 and hTDP43 ALS‐associated mutations. Lane 1–2: *E49‐Gal4* driver. Lane 3–4: *UAS‐hTDP43* transgenic fly. Lane 5–6: *E49‐Gal4 > UAS‐hTDP43* experimental fly. (f) Lane 1–2: *E49‐Gal4* driver. Lane 3–4: *E49‐Gal4 > UAS‐hTDP43* ^*M337V*^ experimental fly. Lane 5–6: *UAS‐hTDP43* ^*Q331K*^. Lane 7–8: *E49‐Gal4 > UAS‐hTDP43* ^*Q331K*^ experimental fly. (h) Negative geotaxis assay of 7‐day‐old *E49 > hTDP43* flies. The *y*‐axis indicates % of flies crossing an 8‐cm threshold. The *x*‐axis indicated time in seconds after flies were tapped down

Given the stability of MN gene expression across age, we hypothesized that *dMMP1* transcript was increasing in response to physiological changes associated with aging, and not just accumulating in a chronologically driven manner. To test this, we repeated quantification experiments on flies that were exposed to different modulators of the aging process. One robust aging intervention is a dietary restriction, flies that eat fewer protein‐derived calories live longer. We evaluated the relative amount of *dMMP1* transcript in wild‐type flies reared on a 1× or 2× yeast diet for 14 days via qRT–PCR (Figure 3a) and Western immunoblot (Figure 3b). These assays revealed that flies raised on the 2× diet demonstrated increases in both *dMMP1* transcript levels and dMMP1 protein levels compared to flies raised on the 1× diet.

Reduced insulin signaling has also been shown to extend lifespan through mechanisms that are largely nonoverlapping with dietary restriction (Min et al., 2008). We tested whether reduced insulin signaling in MNs would be sufficient to limit the accumulation of dMMP1 in MNs. We used qRT–PCR to quantify the amount of *dMMP1* transcript in the thoracic ganglion of aging flies expressing a dominant negative insulin receptor (INR(DN)) under the control of the *D42‐Gal4* motor neuron driver (Figure 3c). Compared to driver and transgene controls, the *D42 > INR(DN)* flies did not show the expected dMMP1 increase 21 days after eclosing. A similar experiment performing the whole‐body (*Act5C‐Gal4* driver) RNAi of the insulin receptor substrate gene *Chico* did not show the same effect (Figure S3). Although we confirmed that *Act5 > Chico* ^*RNAi*^ was reducing *Chico* transcript abundance by less than 50% (Figure S4), this effect may not be strong enough in motor neurons to slow down the accumulation of dMMP1 after 21 days.

We also tested the effect of increased temperature (which shortens lifespan in flies (Miquel et al., 1976)). Flies were reared at 25 or 32°C for 3 or 10 days. qRT–PCR measurements of transcript levels for each duration were compared across temperatures. No detectable difference was seen between 25 and 32°C after 3 days. However, a significant increase in transcript level was detected between the temperatures after 10 days (Figure 3d). Protein levels were evaluated for a 10‐day temperature difference via Western blot (Figure 3e). A moderate (but not statistically significant) increase in *dMMP1* was seen in the 32°C condition.

Disease‐causing alleles can also cause the early manifestation of aging phenotypes. Human Tar‐DNA binding protein 43 (hTDP43) protein has been implicated in the molecular pathophysiology of ALS. It is found in protein inclusions in the affected nervous tissue of patients, and several mutations have been linked to the disease (Sreedharan et al., 2008). Due to toxicity associated with these transgenes, we overexpressed wild‐type hTDP43 with the *E49‐Gal4* driver (a limited MN driver) that resulted in viable adult flies for analysis. Immunoblot analysis of dMMP1 protein levels in thoracic ganglion tissue from flies overexpressing wild‐type hTDP43 showed dramatically increased levels of dMMP1 protein (Figure 3f). We performed similar overexpression experiments with mutant forms of hTDP43 (hTDP43‐M337V and hTDP43‐Q331K) that have been linked to familial ALS and found a significant increase in dMMP1 protein as well (Figure 3g; Lanson et al., 2011). The flies manifested severe motor defects during negative geotaxis (“wall climbing”) assays after overexpression of wild‐type hTDP43 protein (Figure 3h) as well as mutant forms (not shown). This was consistent with reports of the toxic effects of hTPD43 on fly neurons reported by other laboratories (Ishiguro et al., 2017).

Given the potential link between hTDP43‐induced dMMP1 accumulation and motor dysfunction, we tested if reducing the amount or activity of dMMP1 in an *E49 > hTDP43* context would be sufficient to rescue motor behavior deficits. We found that co‐expressing a transgene harboring an inhibitor of dMMP1 activity, the *Drosophila* tissue inhibitor of metalloproteinase 1 (dTIMP1), in the same motor neurons with hTDP43 was insufficient to rescue deficits in negative geotaxis (Figure S6A). We also expressed the deleterious *E49 > hTDP43* expression in a heterozygous null allele background of *dMMP1* (Figure S6B), and this failed to rescue the behavior phenotypes suggesting that the relationship between hTDP43 and motor defects involves other mechanisms (see Discussion).

### 2.4 Negative geotaxis after overexpression of dMMP1 in a subset of motor neurons

**Figure 4:**
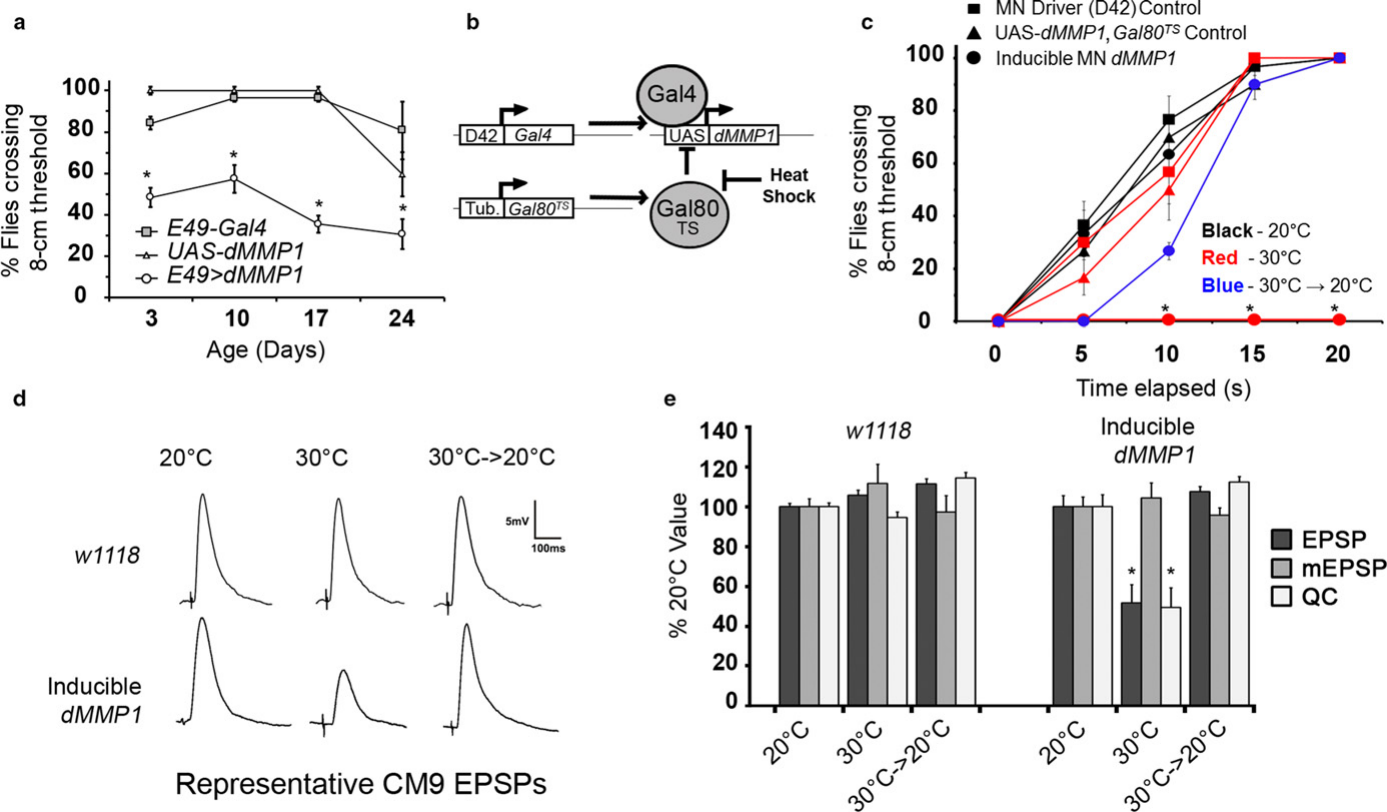
Motor behavior effects of dMMP1 expression in motor neurons. (a) Negative geotaxis assay of flies constitutively expressing dMMP1 in a subset of motor neurons. Square—E49‐*Gal4* driver control. Triangle—UAS‐*dMMP1* transgene control. Circle—E49 > *dMMP1*. *Y*‐axis shows the percentage of flies in the vial that were able to climb to an 8‐cm threshold after 10 s. The *x*‐axis shows the age of the tested flies. Error bars = 1*SD*. *p <* *.05 via one‐way ANOVA (*df* = 2, *F* = 33.64). (b) Scheme for heat‐inducible control of *dMMP1* expression in all motor neurons. White squares show genetic elements, and gray circles show their gene products. (c) Negative geotaxis assay of inducible dMMP1 flies (3 days posteclosion). Squares—D42‐*Gal4* MN driver controls; Triangles—UAS‐*dMMP1*,Tub‐*Gal80* ^*TS*^ transgene controls; D42 > *dMMP1,* Tub‐*Gal80* ^*TS*^ inducible flies. Black—20°C (restrictive temperature); Red—30°C (dMMP1 permissive temperature, 20 hr); Blue—30°C→20°C switch (12 hr dMMP1 permitted, followed by 24‐hr recovery in nonpermissive temperature). Error bars = 1*SD*. *p < .05 *t* test pairwise comparison between inducible flies after heat shock and after recovery. (d) Representative traces of CM9 EPSPs under different dMMP1 induction conditions. (e) Electrophysiology of evoked CM9 MN neurotransmitter release, as measured by excitatory postsynaptic potential (EPSP). The *y*‐axis indicates the % change relative to the baseline (“20°C”). The *x*‐axis indicates temperature (“20°C”—dMMP1 restricted, “30°C” 12 hr after dMMP1 induction, and “30°C→20°C” 24‐hr recovery postinduction). Bar color indicates the EPSP, miniature EPSP (mEPSP), and Quantal Content (QC). One‐way ANOVA of *w1118* flies detected a small, but significant effect of temperature on QC (*p *<* *.000068, *F* = 15.68, *df* = 23). One‐way ANOVA of inducible dMMP1 flies confirmed the large QC reduction seen after dMMP1 induction was highly significant (*p *<* *.00001, *F* = 48.16, *df* = 23)

Because *dMMP1* expression in motor MNs correlates temporally with declines in behavior, we conjectured that *dMMP1* could be directly causing motor deficits to appear in old age. To test this, we decided to overexpress *dMMP1* with various pan‐neuronal and MN specific Gal4 drivers. Unfortunately, these turned out to be universally embryonic lethal (Table 1). This was unsurprising given the proteolytic activity of dMMP1 and the posited role of dMMP1 and dMMP2 in synaptogenesis and axon fasciculation/bundling (Dear et al., 2016; Miller et al., 2008). Fortuitously, the *E49‐Gal4* driver (a limited MN driver) was nonlethal and allowed the adult flies to eclose. Mild motor defects were observed in eclosed adults, so we performed negative geotaxis experiments on *E49‐Gal4, UAS‐dMMP1* flies. Experimental flies were deficient at self‐righting and climbing compared to control flies (Figure 4a). The effect became progressively stronger with age as well, possibly due to either accumulating damage from dMMP1 overexpression or from the added effect of the endogenous dMMP1 allele increasing expression with age.

**Table 1:** Summary of UAS‐*dMMP1* transgene effect in combination with different neuronal drivers. The expression of dMMP1 was embryonic lethal when driven pan‐neuronally. Crosses between the driver line and transgene line did not yield larvae. Restricted neuronal expression through the E49‐Gal4 driver (which expresses in neurons controlling feeding behavior) were viable and could be maintained indefinitely after recombination

*Gal4* driver name	Tissue expression	UAS‐*dMMP1* phenotype
*Elav C155*	All neurons	Embryonic lethal
*D42*	Motor neurons	Embryonic lethal
*D42,TubGal80* ^*TS*^	Motor neurons (Inducible)	Severe ataxia on induction^a^
*C380*	Motor neurons	Embryonic lethal
*OK6*	Motor neurons	Embryonic lethal
*E49*	Limited set of motor neurons	Mild ataxia, self‐righting defects

a Inducible *D42 > dMMP1* was also larval and adult lethal if allowed to remain on for >16 hr.

### 2.5 Negative geotaxis in after dMMP1 induction in all motor neurons

Because of the limited expression of the *E49*‐Gal4 driver and its expression in peripheral sensory neurons (Gordon & Scott, 2009), we created a fly line with the *D42‐Gal4, UAS‐dMMP1* system repressed by a temperature‐sensitive Gal80 (*UAS‐Gal80* ^*TS*^). The logic of the system is outlined in Figure 4b. Briefly, this line only activates MN *dMMP1* expression after being heat shocked at 30°C. We repeated the negative geotaxis test in this line in three conditions: before induction (20°C), 20 hr after induction (30°C), and 24 hr after recovery at 20°C following a 12‐hr induction (Figure 4c). The induction of *dMMP1* in all motor neurons created a dramatic phenotype, where initially the flies are impaired from climbing, and (if continued for 20 hr or more) are almost completely paralyzed. Notably, if these flies are allowed time to recover immediately after the climbing deficits become obvious but before becoming complexly unable to climb (~12 hr), they recover almost completely. This reversible phenotype implies that the defects seen in negative geotaxis are not due to cell death but due to a temporary problem in neuromuscular performance. A representative example of the acute (20 hr) deficit can be seen in Movie S2.

### 2.6 Electrophysiology of CM9 neuron after dMMP1 induction

Our laboratory has previously developed a NMJ model in the adult fly using the NMJs on the Cibarial muscle 9 (CM9) that allows for quantal analysis of the evoked excitatory postsynaptic potential (EPSP) using electrophysiology (Rawson et al., 2012). In addition to quantifying the amount of neurotransmitter released by the nerve terminal, referred to as quantal content (QC), this approach also allows us to directly measure the excitability of the muscle. We used the CM9 NMJ model to investigate the possibility that the motor defects manifested in the *dMMP1* inducible flies were caused by problems in synaptic transmission at the NMJ. We observed significant reductions in the size of the EPSP after induction of *dMMP1* in 3‐day‐old animals heat shocked for 16 hr. Representative traces of CM9 EPSPs from these analyses are shown in Figure 4d, and quantification is shown in Figure 4e. Because we did not observed a change in the amplitude of the EPSP resulting from the release of a single synaptic vesicle (mEPSP), we conclude that the reduction in the EPSP is due entirely to the reduction neurotransmitter release (QC). Consistent with negative geotaxis experiments, neurotransmission recovered after 24 hr of stopping transgenic *dMMP1* expression. These results suggest that acute *dMMP1* expression reversibly impedes the release of neurotransmitter from the presynaptic nerve terminal of the NMJ.

### 2.7 dMMP1 localizes to the NMJ synapse but does not cause morphological changes after overexpression

**Figure 5:**
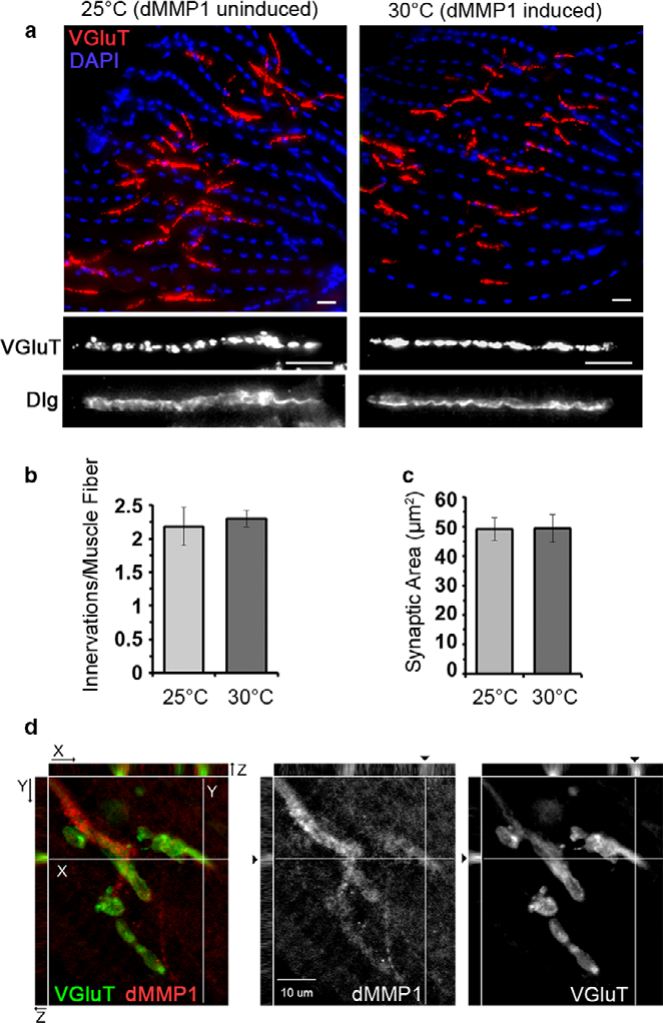
Immunostaining for morphological analysis of synapses at the CM9 NMJ. (a) Representative images of immunostained CM9 dissections before and after induction of transgenic dMMP1. Top panel is 40× magnification showing a whole representative CM9 muscle, and bottom panel is a higher magnification image of a representative bouton. (b) Quantification of number of boutons counted per CM9 muscle fiber. (c) Quantification of total synaptic volume as measured by masking total vGluT signal from boutons. (d) Three‐dimensional reconstruction of a CM9 NMJ showing localization of dMMP1 around axons (left panel) and in the presynaptic nerve terminal (right panel)

Given the role in extracellular matrix remodeling and synaptogenesis established for dMMP1, we performed immunofluorescent microscopy of the CM9 NMJ before and after induction of transgenic *dMMP1* to determine whether dMMP1 expression leads to remodeling of the NMJ. To visualize for the pre‐ and postsynaptic apparatus, we used antibodies to the synaptic vesicle protein VGluT and the postsynaptic PSD‐95 homologue Discs‐large (DLG) (Figure 5a). We observed no change in the number of boutons per muscle fiber or synaptic volume (Figure 5b,c). We also observed no evidence of “synaptic footprints” caused by retraction or die‐back of the motor neuron. These observations demonstrate that the increased expression of dMMP1 does not result in remodeling of the NMJ. This is consistent with previous analysis of CM9 NMJ morphology across age (Mahoney et al., 2014).

Because of the strong effect of dMMP1 overexpression on presynaptic function, we investigated whether dMMP1 might localize at the CM9 NMJ. We performed immunofluorescent microscopy of CM9 NMJs to ascertain whether dMMP1 can be detected at the presynaptic terminal. Near the NMJ, we found detectable dMMP1 in axons, especially at branch points, glial cells, and throughout the muscle. We also could detect dMMP1 at the NMJ, although at much lower levels than what we observe in the axon. To confirm that this staining was presynaptic, we generated three‐dimensional reconstructions of serially sectioned NMJs allowing us to determine that at least a portion of the dMMP1 immuno‐reactivity observed at the NMJ is found within the presynaptic nerve terminal as defined by VGluT staining (Figure 5d). It should be noted that we do not observe perfect co‐localization of dMMP1 and VGLuT, which is expected as dMMP1 has not been found on synaptic vesicles.

### 2.8 Negative geotaxis and survival after abrogation of dMMP1 in all motor neurons

**Figure 6:**
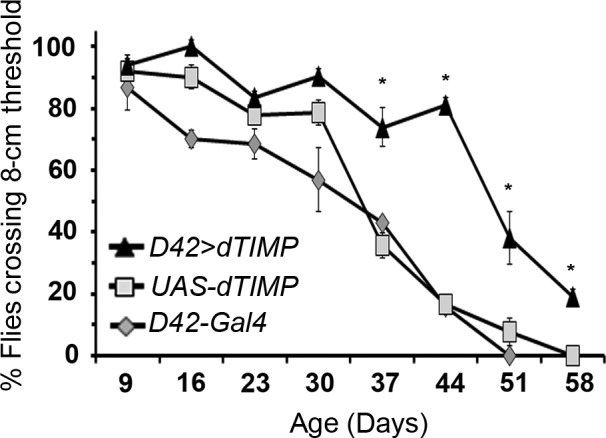
*Drosophila* tissue inhibitor of metalloproteinases (dTIMP) rescue of motor deficits. Negative geotaxis assay of flies constitutively expressing *dTIMP* in all motor neurons. Diamond‐ D42‐*Gal4* driver control. Square—UAS‐*dTIMP* transgene control. Triangle—D42 > *dTIMP*. The *y*‐axis shows the percentage of flies in the vial that were able to climb to an 8‐cm threshold after 10 s. The *x*‐axis shows the age of the tested flies. Error bars = 1*SD*. *p <* *.05 via one‐way ANOVA

Given that dMMP1 overexpression is sufficient to cause motor and electrophysiological deficits, we predicted that experimentally reducing the amount or activity of *dMMP1* in old animals could ameliorate declines in motor behavior and locomotion. We tested a publicly available *dMMP1* RNAi line (Bloomington #31489), but it did not seem to reduce the amount of *dMMP1* transcript detectable by qPCR (Figure S4). Therefore, we decided to overexpress the transgene harboring dTIMP (the inhibitor of MMPs) in all motor neurons and determine whether this manipulation of MMP activity could rescue the motor declines observed with age. In contrast to overexpression of *UAS‐dMMP1*, overexpression of *UAS‐dTIMP* in motor neurons using the *D42‐Gal4* was not lethal and eclosed adults had no obvious phenotypic defects. Negative geotaxis experiments on driver and transgene controls as well as *D42 > dTIMP* flies showed no significant differences in climbing behavior at early ages, but demonstrated a protective effect of dTIMP 37 days posteclosion that was maintained into old age (Figure 6). The protective effect of dTIMP on motor function led us to hypothesize that *dTIMP* overexpression in MNs might be sufficient to prolong *Drosophila* lifespan. We observed that mean and median lifespans were not significantly different between controls and *dTIMP‐*overexpressing flies, indicating that delaying the age‐dependent motor deficits by dTIMP overexpression in MNs is not sufficient to extend lifespan (Figure S5, Table S5).

## 3 DISCUSSION

### 3.1 Transcriptomics of motor neurons

Age is the most important predictor of neurodegenerative diseases such as Alzheimer's disease, Parkinson's disease, and amyotrophic lateral sclerosis (ALS). However, we still do not know how age interacts with disease alleles and environmental insults to cause neurodegeneration. We predicted that neuron‐specific changes in the transcriptome driven by aging could represent genes that are key factors in the pathogenesis of neurodegeneration. We tested this by querying the transcriptome of motor neurons from *D. melanogaster* across age. Differential transcriptomics approaches have been previously used to investigate the effects of aging on gene expression. Some of these studies have been performed on whole animals or whole organ systems (Baumgart et al., 2014; Cannon et al., 2017; Fraser et al., 2005; Lee et al., 2000; Lu et al., 2004; Pletcher et al., 2002). These approaches have yielded insights into the aging process. However, due to noise from heterogenic tissue in these preparations, it is possible that cell‐ and tissue‐specific dysfunctions are occluded in these approaches. Our analysis limited itself to one highly homogenous postmitotic cell type, allowing us to connect changes in the transcriptome to changes in physiological function. Our analysis revealed that in MNs surprisingly, few transcripts change significantly during aging and that most genes, such as the neurotransmitter synthesis genes associated with cell identity, were expressed stably across the ages tested (Figure 1b). It is possible we may have observed more substantial changes if we had profiled across a wider aging window, although we found it difficult to generate consistent probes from old motor neurons. Additionally, our statistical analysis was conservative and so may be underestimating the number of transcripts significantly altered with age. Alternatively, MNs in *Drosophila* could have a particularly stable transcriptome due to their postmitotic state and highly stereotyped neuronal identity that is strictly maintained.

### 3.2 MMPs represent susceptibility factors for neurodegeneration

Our laboratory and others have previously shown that *Drosophila* experience age‐dependent declines in motor behavior, analogous to the declines seen in mammals. We found that the gene encoding the *Drosophila* homolog of the matrix metalloproteinases 1 (*dMMP1*) consistently and dramatically changed expression with age. Because the increases in *dMMP1* correlate with declines in motor behaviors, we tested if *dMMP1* expression alone was sufficient to recapitulate behavioral deficits. We found that by driving *dMMP1* in a small subset of MNs constitutively or by induced overexpression in all MNs, we could elicit reversible climbing deficits (Figure 4a‐c). Consistent with these observations, we found that neurotransmission at the NMJ was reversibly disrupted in response to the same acute overexpression paradigm (Figure 4d,e). This correlation implies that an initial mechanism of action by which dMMP1 impairs motor function in old animals is by disrupting the release of neurotransmitter at the NMJ. A decrease in neurotransmission at the CM9 NMJ was previously described at old ages (Mahoney et al., 2014).

It is unclear how *dMMP1* alters synapse function. Recent work has implicated MMP activity as important for synaptogenesis during larval development (Dear et al., 2016). Our ability to detect dMMP1 protein around axons and near boutons in the adult NMJ is consistent with these findings although we observe no effect of increased dMMP1 expression on synaptic innervation or growth in the adult. Dear et al. also reported that MMPs could inhibit neurotransmission by altering *Wnt* signaling. Review of our microarray data found no evidence that *wingless* signaling was altered during aging (Table S2).

*MMP* upregulation with age may have important implications for neurodegenerative disorders in humans. A recent paper (Kaplan et al., 2014) demonstrated that one of the key molecular differences between MNs that are vulnerable to ALS progression and those that are not is the expression of MMP9. These researchers showed that MMP9 was highly expressed in subpopulations of motor neurons that are more susceptible to degeneration in the presence of a human ALS mutation (SOD1‐G93A). Tantalizingly, we found that by overexpressing hTDP43 in neurons, we were able to dramatically accelerate the rate of dMMP1 accumulation in the thoracic ganglion (Figure 3f,g). We cannot rule out the possibility that this is a noncell autonomous response, as qRT–PCR from sorted *E49 > GFP, hTDP43* MNs only showed a small nonsignificant increase in the dMMP1 transcript (data not shown), or that this is due to changes in protein turnover. Regardless, the increase in MMP protein in response to overexpression of TDP43 suggests a potential pathogenic mechanism for ALS.

Our data also illustrate important differences between the effects of aging and neurodegenerative disease‐causing mutations on motor function. Under both conditions, aging and neuronal overexpression of hTDP‐43, we observe that the flies have pronounced motor deficits and elevated levels of dMMP1. We find that in the case of aged flies, inhibition of dMMP1 activity reduces the observed deficits in motor function supporting the model that increasing *dMMP1* expression in motor neurons is an important contributor to the declines in motor function observed during aging. In contrast, a similar inhibition of dMMP1 activity did not improve motor function in flies expressing *hTDP‐43*. We interpret this result to indicate that the hTDP‐43‐expressing flies are succumbing to pathologic mechanisms other than those associated with increasing dMMP1. Perhaps this is not surprising given the function of TDP‐43, a ubiquitously expressed RNA‐binding protein involved in RNA processing and nuclear export that binds to a broad range of mRNAs (Buratti & Baralle, 2010; Tollervey et al., 2011). It should be noted that to date, no matrix metalloproteinase mRNAs have been reported to require TDP‐43 for normal expression. Finally, the observation that dMMP1 protein levels are increased in hTDP‐43‐expressing flies, but does not contribute to motor dysfunction, suggests that potentially dMMP1 function is altered in the TDP‐43‐overexpressing neurons. We currently do not know what isoforms of dMMP1 are being expressed during aging and in response to TDP‐43 overexpression but it is possible that these two conditions have very different isoform expression patterns. Given the important role of MMP‐9 in the SOD‐1 model of ALS (Kaplan et al., 2014), these data highlight the difficulty in assigning specific genetic mechanisms to the pathogenesis of neurodegeneration in the TDP‐43 models of ALS.

### 3.3 Metalloproteinases as Antagonistically Pleiotropic Genes

The theory of antagonistic pleiotropy predicts that organisms will carry genes that give a benefit at a young age, but exact a fitness cost after reproduction (Williams, 1957). Matrix metalloproteinase genes are excellent candidates—they are required to shape the tissues of the developing metazoan into the forms required to survive and reproduce. In *Drosophila*, dMMP1 is required throughout the peripheral nervous system for normal development including axon fasciculation and the remodeling of both dendrites and nerve terminals (Dear et al., 2016; Meyer & Aberle, 2006; Miller et al., 2008). After development, the dMMP1 expression is clamped down in the adult peripheral nervous system, including the MNs. We now show that the repression of *dMMP1* expression within the MNs is gradually lost during aging resulting in deleterious consequences for the organism.

Mammalian MMPs such as MMP9 are required for normal tissue remodeling during development of the nervous system and other tissues (Ethell & Ethell, 2007; Page‐McCaw et al., 2007). This includes MMP9, which is required for circuit and synaptic reorganization and plasticity within the adult brain (Wiera et al., 2013). But MMPs have also been identified as potential molecular players in neurodegenerative disorders, including ALS, PD, and AD (Beuche et al., 2000; Kiaei et al., 2007; Lim et al., 1996; Rosenberg, 2009). MMPs are also known to be upregulated in aging skin and metastatic tumors (Fingleton, 2006; Jenkins, 2002). And the expression of MMP3, MMP1, and MMP13 is part of the senescence‐associated secretory phenotype (SASP) in mammalian cells (Coppe et al., 2010; Parrinello et al., 2005).

How does aging result in increased *dMMP1* expression? One possibility is aging alters dMMP1 transcription via the oxidation of promoter, enhancer, or repressor DNA sequences (Ghosh & Mitchell, 1999; Lu et al., 2004). However, in our experiments with *chico* ^RNAi^ (which is predicted to increase antioxidant pathways) and the free radical generator paraquat, we did not observe effects on dMMP1 accumulation (Figure S3B). We also considered the possibility that dMMP1 increase was mediated through a JNK pathway inflammatory response. A review of our microarray data did not detect other genes known to be regulated by JNK signaling (Table S2). Further research will be needed to explore whether these or other epigenetic mechanisms are responsible for reactivation of MMPs during aging.

## 4 EXPERIMENTAL PROCEDURES

For a detailed description of *Drosophila* husbandry, RNA purification and amplification, qRT–PCR, Western blots and immunostaining, electrophysiology, negative geotaxis, and statistical analysis see Appendix S1—Methods. Primers used for qRT–PCR are listed in Table S4, and efficiency curves for primers are shown in Figure S1. Primary antibodies used and their concentrations are listed in Table S4.

### 4.1 Microarray hybridization and analysis

Cy3‐labeled cDNAs were hybridized to an Agilent custom *Drosophila* microarray (8 × 60 K format, Design ID: 065755) at the UTHSCSA Genomics Resource Core. Agilent Feature Extraction 11 software was used to convert the image signal to numerical values for each feature on the microarray. Data were analyzed using Agilent GeneSpring™ software, which also mapped the feature signal to the gene represented by that feature. Analysis workflow was initiated by loading the correct microarray technology, then adding the sample files and verifying through standard quality control measures that arrays were properly hybridized. Normalization was carried out via 75th percentile normalization (the default setting for Agilent single‐color technologies). Samples were assigned a parameter “AGE” with values of “Young,” “Pre‐ADP,” and “Post‐ADP.” Analysis included principal component analysis (PCA), unbiased hierarchical clustering of averaged feature intensity, volcano plot analysis of differentially expressed genes across conditions, ANOVA tests across multiple conditions. All tests were carried out under Benjamini–Hochberg correction for multiple testing. Full data set was deposited to NCBI GEO under accession number GSE106632.

## AUTHOR CONTRIBUTIONS

J.A. designed experiments; performed molecular biology, microarray analysis, negative geotaxis experiments, immunostaining, survival analysis; and wrote the manuscript. R.E.M. performed negative geotaxis experiments and electrophysiology. B.A.E. designed experiments and wrote the manuscript.

## CONFLICT OF INTEREST

None declared.
