# Supplementary material for: Transcriptomics of aged Drosophila motor neurons reveals a matrix metalloproteinase that impairs motor function
Source: Aging Cell. 2018 Feb 7;17(2):e12729. doi: 10.1111/acel.12729 (PMC5847883; doi:10.1111/acel.12729)
Supplement: Supplementary file 12 [file ACEL-17-e12729-s012.docx]

*Fly Husbandry*

The *Drosophila* strain *w^1118^* was used as a control where indicated. *Gal4* driver lines and all transgenic flies were backcrossed to *w^1118^* for at least seven generations for geotaxis experiments. Flies used for longevity experiments were backcrossed at least ten generations. Unless otherwise indicated *Drosophila* stocks were maintained at 25 °C, on a 12-h light/dark cycle, at constant 50% humidity using standard sugar/yeast/agar (SYA) media. Animals used in experiments that had age as a variable were flipped every 48 hours to a new vial and any dead animals were removed. Full genotypes for all used lines are indicated in **Supplemental Table 3**.

*RNA Purification and Amplification*

Cells from the dissected lower thorax of adult *Drosophila* flies expressing GFP in all motor neurons were dissociated via 30 minute digestion in 1mg/mL collagenase type I (Thermo Fisher Scientific; Waltham, MA). Dissociated cells were then run through a FACS Aria cell-sorter (BD BioSciences; East Rutherford, NJ) using a 70µm nozzle and 4-way purity setting at the UTHSCSA Flow Cytometry Core. Cells were sorted directly into Qiagen RNEasy Micro Kit buffer RTL-Plus (Qiagen; Hilden, Germany) with 1% (v/v) 2-mercaptoethanol (Sigma-Aldrich; St. Louis, MO). Cells in RTL were then run through the standard kit protocol. cDNA amplification and labeling was performed using a MessageAmp™ II Amino Allyl aRNA Kit (AM1753, Thermo Fisher Scientific). Sample amplification was verified by NanoDrop™ (Thermo Fisher Scientific) and BioAnalyizer™ (Agilent Technologies; Santa Clara, CA) at the UTHSCSA Genomics Resource Core. Samples were coupled to Cy3 NHS Ester fluorescent dye (GE Healthcare; Little Chalfont, UK) and purified prior to microarray hybridization.

*Western Blot*

*Drosophila* tissues were homogenized in 2X SDS buffer containing 1% (v/v) 2-mercaptoethanol (100µL per 7 dissected thoracic ganglions or 1 whole fly) and boiled for 10 minutes. 5μL of homogenate were loaded into a 4-15% TGX Mini-Protean™ Precast gel (Bio-Rad, Hercules, CA) and run for 1 hr at a constant 100V in standard SDS-glycine buffer. Proteins were transferred to nitrocellulose in 4°C sodium tetraborate buffer at a constant 350 mA for 45 minutes, then blocked in 5% (w/v) BSA for 30 minutes. Detection of bands was accomplished through reacting HRP-conjugated secondary antibodies (1:5000 in 3%BSA) and Novex™ ECL (Thermo Fisher Scientific). Quantitation was performed using ImageJ software (National Institutes of Health).

*Immunostaining*

Adult CM9 muscles were dissected after fixing in 4% paraformaldehyde for 15 minutes, then washed twice in 1x PBT to remove the fixative. Tissues were blocked with Image-iT FX Signal Enhancer (Thermo Fisher Scientific) for 30 minutes, then rinsed once in PBT and incubated with primary antibody (1:100 dilution) overnight at 4°C. After three 1X PBT washes (10 minutes), tissues were incubated in Alexa Fluor (Thermo Fisher Scientific) conjugated secondary antibodies (1:500) for 10 minutes, then washed three times with 1X PBT. Tissues were then mounted in Vectashield with DAPI (Vector Laboratories; Burlingame, CA). Synaptic volumes were quantified using the method described in Mahoney, Rawson et al. 2014. For three dimensional reconstructions, fixed and stained CM9 NMJs were serially sectioned at 0.2 um per section and reconstructed using Slidebook software (3I, Denver, CO).

*Quantitative RT-PCR*

Purified total RNA from sorted motor neurons was reverse transcribed into cDNA by SuperScript™ iii (Thermo Fisher Scientific) polymerase using random hexameric primers. Applied Biosystems SYBR Select (Thermo Fisher Scientific) was used as the detection dye and reactions were run on an Applied Biosystems 7500 Fast (Thermo Fisher Scientific) qPCR system. Reference gene was tubulin 84B for all experiments. Relative transcript quantities were determined by the ∆∆Ct method (except for *LacZ* amplification, where a modified ∆∆Ct method accounting for lower primer pair amplification efficiency was used). Primer efficiency curves are shown in Supplemental Figure 1.

*Negative* *Geotaxis*

For negative geotaxis experiments virgin female flies from tested genotypes were sorted upon eclosion and aged in standard husbandry conditions listed above. Flies were tested for self-righting and climbing speed by transferring to plastic vials containing no food and with an 8cm mark. Flies were tapped down with 3 mock trials prior to actual experimental trials. All geotaxis cohorts were tested within an hour of each other and all geotaxis experiments were performed in the morning. Climbing was recorded with a digital video camera and timer and analyzed after all trials were completed.

*Electrophysiology*

CM9 evoked post-synaptic potentials were recorded according to methods previously described ([Mahoney et al. 2014](#_ENREF_28)). Dissections and recordings in this manuscript were performed in a modified HL3 solution (containing, in mM: 70 NaCl, 5 KCl, 10 NaHCO_3_, 5 trehalose, 115 sucrose, 5 HEPES, 1 CaCl_2_, 3 MgCl_2_)

*Data and statistics*

Data for WB quantification, qRTPCR, negative geotaxis, and synapse area and number quantification are reported as the average ± SEM.  Significance was determined using Student’s T-test for pairwise comparisons and one-way ANOVA with a Bonferroni correction for all multiple comparisons.  All statistical analyses were performed using Prism 6 (Graphpad). *F* statistics, *p* values and degrees of freedom (*df*) are indicated in figure legends of applicable experiments.
