# Supplementary material for: Transcriptomics of aged Drosophila motor neurons reveals a matrix metalloproteinase that impairs motor function
Source: Aging Cell. 2018 Feb 7;17(2):e12729. doi: 10.1111/acel.12729 (PMC5847883; doi:10.1111/acel.12729)
Supplement: Supplementary file 15 [file ACEL-17-e12729-s015.docx]

**Supplemental Figure 1**. qPCR Primer Validation and efficiency. Critical threshold value was plotted against a dilution series of whole fly cDNAs known to contain the tested transcript. Efficiency was derived from the slope of the line. (**A**) *dMMP1* qPCR primers. (**B**) *LacZ* qPCR primers. (C) *dMMP2* primers. (D) *Chico* primers.

**Supplemental Figure 2**. Map of LacZ enhancer trap line with insertion in dMMP1. An isoform of *dMMP1* is shown to approximate scale. Scale bar indicates 1 kilobase distance. Exons are shown as boxes, with grey indicating 5’ and 3’ UTRs. Introns are shown as black lines between exons.

**Supplemental Figure 3**. Western immunoblot of chico^RNAi^ flies and flies treated with paraquat. (A) w1118 control flies and flies driving a UAS-chico^RNAi^ transgene in all cells (through the pan-cellular Act5-*Gal4* driver) 35 days post-eclosion. (B) w1118 flies were treated with vehicle (ethanol) or paraquat (sublethal, 10mM) for 7 days (from 7 to 14 days post-eclosion).

**Supplemental Figure 4**. Knockdown validation of RNAi lines. (**A**) Attempted knockdown of *dMMP1* via Act5>*dMMP1*^RNAi^ did not show a decrease in transcript. Fold change is shown relative to transgene control. (**B**) Knockdown of *Chico* via Act5>Chico^RNAi^ yielded of a statistically significant (*p*<0.05, Student’s t-test) transcript reduction of about 50%. Fold change is shown relative to transgene control.

**Supplemental Figure 5**. Mortality curve of D42>*dTIMP* flies (and driver/transgene controls). Gray line- D42-*Gal4* driver control. Broken line- UAS-*TIMP* transgene control. Black line- D42-*Gal4*→UAS-*TIMP*. Analysis of all three groups was performed via the Log-rank (Mantel-Cox) test and found significant difference in curve shape (Chi square = 9.111, df=2, *p* = 0.0105). Explicit mortality curve data can be found in **Supplemental Table 5**.

**Supplemental Figure 6**. hTDP43 rescue experiments. (**A**) Simultaneous overexpression of TIMP and hTDP43 with E49 driver was insufficient to rescue negative geotaxis defects observed in *E49>hTDP43* flies. (**B**) Overexpression of hTDP43 in a *dMMP1* heterozygous background (*dMMP1*^-/+^ ) was insufficient to rescue negative geotaxis defects observed *in E49>hTDP43* flies. Error bars = 1 SEM. Significance tested by Student’s t-test comparing *E49>hTDP43* to the corresponding rescue condition.

**Supplemental Movie 1**. Negative geotaxis movies of control and inducible flies at non-permissive temperature (20°C).

**Supplemental Movie 2**. Negative geotaxis movies of control and inducible flies after 20 hours at permissive temperature (30°C).

**Supplemental Table 1** – Drosophila genes and isoforms curated for custom microarray enrichment. We downloaded all genes and isoform from Flybase that were functionally annotated as either ion channels (Sheet 1) or calcium-binding proteins (Sheet 2). The purpose was to enhance our ability to detect more subtle changes in ion channel expression and potentially do transcript and isoform analysis. Data analysis took into account the fact that multiple features mapped to the same gene and was accounted for in multiple testing correction.

**Supplemental Table 2** – GeneSpring output for ANOVA test across all conditions (“age” parameter- samples grouped into young, pre-ADP or post-ADP). Second sheet shows summary of most significantly changing features and genes that may influence neurotransmission in the older fly.

**Supplemental Table 3** – *Drosophila melanogaster* stocks used in this study.

**Supplemental Table 4** – Oligonucleotide primer sequences and primary antibodies used in this study.

**Supplemental Table 5** – Comprehensive mortality data for **Supplemental** **Figure 5**.
